# Exploring mental health professionals’ emotional responses with individuals diagnosed with antisocial personality disorder or psychopathy: a scoping review

**DOI:** 10.3389/fpsyg.2025.1501273

**Published:** 2025-06-25

**Authors:** Gabriele Lo Buglio, Leonardo Zaninotto, Renan Göksal, Uyangakhishig Bayasgalan, Alice Barsanti, Brad D. Booth, Nicola Carone, Anmar Mighri, Alessandro Miola, Laura Muzi, Vittorio Lingiardi, Marco Solmi, Tommaso Boldrini

**Affiliations:** ^1^Department of Dynamic and Clinical Psychology, and Health Studies, Faculty of Medicine and Psychology, Sapienza University of Rome, Rome, Italy; ^2^Local Health Unit No. 6 ("Euganea"), Department of Mental Health, Padua, Italy; ^3^Department of Developmental Psychology and Socialization, University of Padova, Padua, Italy; ^4^Digital Commons Lab, Fondazione Bruno Kessler, Trento, Italy; ^5^Division of Forensic Psychiatry, Department of Psychiatry, University of Ottawa, Ottawa, ON, Canada; ^6^The Royal Ottawa Mental Health Centre, Ottawa, ON, Canada; ^7^Department of Systems Medicine, University of Rome Tor Vergata, Rome, Italy; ^8^Department of Psychiatry and Psychology, Mayo Clinic, Rochester, MN, United States; ^9^Department of Neuroscience, University of Padova, Padova, Italy; ^10^Department of Philosophy, Social Sciences, Humanities, and Education, University of Perugia, Perugia, Italy; ^11^Department of Psychiatry, University of Ottawa, Ottawa, ON, Canada; ^12^Department of Child and Adolescent Psychiatry, Charité Universitätsmedizin, Berlin, Germany; ^13^Regional Centre for Treatment of Eating Disorders, and On Track: Champlain First Episode Psychosis Program, Department of Mental Health, Ottawa Hospital, Ottawa, ON, Canada; ^14^Clinical Epidemiology Program, Ottawa Hospital Research Institute, University of Ottawa, Ottawa, ON, Canada; ^15^School of Epidemiology and Public Health, Faculty of Medicine, University of Ottawa, Ottawa, ON, Canada; ^16^Department of Psychology and Educational Science, Pegaso Telematic University, Naples, Italy

**Keywords:** countertransference, therapist emotional response, antisocial personality disorder, psychopathy, scoping review

## Abstract

**Aims:**

To conduct a scoping review of primary research studies on clinicians’ emotional responses (i.e., countertransference) when working with individuals with antisocial personality and/or psychopathy traits or disorders. A secondary aim is to map clinicians’ personal opinions on managing these individuals clinically, as they can influence clinical decision-making.

**Methods:**

A PRISMA-ScR compliant scoping review was conducted. PubMed, Web of Science, and EBSCO/PsycINFO were searched for studies published from 01/01/1990 to 06/12/2023, including clinicians providing clinical care to individuals with antisocial or psychopathy traits or disorders assessed via standardized and validated tools and focusing on their cognitive, emotional and behavioral responses, with no restriction regarding context/location and study design (protocol: https://osf.io/eaquj).

**Results:**

Twelve studies were included, mostly from Europe, mainly focusing on clinicians working with individuals with ASPD. Only two studies addressed psychopathy. Key findings were organized into four concepts: (a) “common emotional responses,” which included nervousness, fear of aggression, detachment, and frustration experienced by the clinician; (b) “willingness to deliver clinical care,” indicating the key role of clinicians’ confidence in their perceived knowledge and skills to provide effective psychological treatment and highlighting that clinicians working in forensic mental health settings exhibit a higher motivation to provide clinical care compared to those in non-forensic settings; (c) “emotional responses related to misogynistic behaviors,” showing additional challenges for assigned female therapists in group therapy; (d) “opinions about clinical management and therapeutic alliance,” emphasizing the crucial role of the clinician’s respect, recognition, and flexible yet firm boundaries.

**Conclusion:**

Individuals with antisocial and psychopathy traits or disorders pose significant challenges in terms of clinicians’ emotional responses, highlighting the need for tailored training and supervision to enhance their competence and confidence. The absence of cohort studies and randomized controlled trials on this subject, along with the limited evidence on psychopathy, warrants for further research.

**Systematic review registration:**

https://osf.io/eaquj.

## 1 Introduction

Since Karl Jasper’s conceptualization of empathic understanding (Jaspers, 1959), classical literature in psychiatry and clinical psychology has considered empathy (“Einfühlung”) and intuitive knowledge as crucial elements for diagnosis, clinical reasoning and treatment. This view highlights the epistemological importance of incorporating both the patient’s and the clinician’s subjectivity into the clinical assessment process (Fuchs, 2010; Stanghellini, 2007). However, in recent decades, it has often been overshadowed by the rise of atheoretical, nosographic, and criteria-based diagnostic approaches, which exclude subjectivity from the assessment process and instead focus on describing symptoms from a third-person perspective (Blashfield, 1984). Such diagnostic manuals have been criticized for their inability to fully capture the specific characteristics of the patient’s experience, which can be better understood by adopting a second-person perspective (i.e., within the intersubjective frame of the therapeutic relationship), where inferences about the other’s experiences, emotions and beliefs can be explored, responded to, discarded, clarified, and confirmed, by both parties (Gupta et al., 2019; Lingiardi and McWilliams, 2015b).

Psychoanalysis has extensively explored the complex intersubjective dynamics that emerge within the therapeutic relationship. One of its central conceptual contributions is the notion of countertransference, defined as the unconscious reaction of the psychoanalyst to the patient’s transference, which—if unrecognized—may hinder the therapeutic process (Freud, 1958). A more general definition considers countertransference as the clinician’s overall reaction—conscious and unconscious, emotional, cognitive, and behavioral—to the patient, which can be used within the therapeutic process to better understand the patient’s interpersonal functioning (Gabbard, 1995; Hayes, 2004).

Although originating from different theoretical frameworks, the concepts of countertransference, projective identification, cognitive interpersonal cycle, or interpersonal complementarity all share the view that our behavior tends to evoke specific and reproducible emotional reactions in those we interact with (Colli et al., 2014). Such emotional reactions can be used within the therapeutic relationship to gather important information about the patient’s personality (Colli et al., 2014; Kernberg, 1965), to affect the therapeutic process (Dahl et al., 2014; Hayes et al., 2011; Markin et al., 2013; Ulberg et al., 2014; Westra et al., 2012), and to influence the development and the maintenance of the therapeutic alliance (Gelso and Hayes, 2007; Hilsenroth et al., 2012; Safran and Muran, 2000). Exploring the complex reactions in therapists and staff may be crucial for refining the diagnostic processes, developing comprehensive and meaningful case formulations, and planning tailored therapeutic interventions (Lingiardi and McWilliams, 2015a).

Exploring therapists’ and staff members’ emotional reactions is particularly relevant when working with patients who experience disruptions in their sense of self, perception of others, and relational patterns, such as those with personality disorders (Kernberg, 1984; Lingiardi and De Bei, 2011). Such individuals tend to elicit intense and challenging emotional reactions (Carsky, 2021), which may affect not only the provision of treatment but also mental health professionals’ confidence and risk of burnout (Holmqvist and Jeanneau, 2006; Rossberg and Friis, 2003).

Among personality disorders, antisocial personality disorder (ASPD) is characterized by a pervasive pattern of disregard for other people’s feelings, often accompanied by violations of social norms (American Psychiatric Association, 2013). The lifetime estimated prevalence of ASPD is 3.6% in psychiatric settings (Zimmerman et al., 2008) and ranges from 1 to 4% in the general population (Werner et al., 2015). Although often regarded merely as a personality construct, individuals with ASPD are at increased risk of developing a range of comorbid mental health conditions, including a fourfold higher risk of mood disorders, a two fold increased risk of anxiety disorders, a thirteen fold risk of substance use disorders, and a seven-to nine fold increased risk of suicide (Alegria et al., 2013; Goodwin and Hamilton, 2003; Werner et al., 2015).

A related construct with partial overlap with the DSM definition of ASPD is psychopathy. Individuals with psychopathy typically display more pronounced interpersonal and affective deficits than those with ASPD. This includes superficial charm, grandiosity, remorse deficits, shallow affect, and callousness (Cleckley, 1988). Moreover, it is rather considered as a neuropsychiatric disorder characterized by low anxiety and low reactivity to stress and punishment (Newman and Brinkley, 1997; Verona et al., 2004), normal executive functioning (Dolan, 2012), and less impulsive and more premeditated acts of violence compared to ASPD (Patrick and Zempolich, 1998). The prevalence of psychopathy is 3% in psychiatric settings, while it is not fully established in the general population (Douglas et al., 1999).

Although no treatment has yet achieved broad consensus or definitive validation for ASPD, several approaches have shown promise. According to the NICE guidelines, group-based cognitive and behavioral interventions should be considered for individuals with ASPD (including those with co-occurring substance misuse) to address impulsivity, interpersonal difficulties, and antisocial behaviors [National Collaborating Centre for Mental Health (UK), 2010]. However, Cochrane reviews consistently underlined the scarcity of high-quality evidence supporting any specific intervention (Gibbon et al., 2010, 2020). Some randomized controlled trials suggest that specialized psychotherapies—such as Schema Therapy (Bernstein et al., 2023) or Mentalization-Based Therapy (MBT) (Fonagy et al., 2025)—may reduce aggression and improve clinical outcomes. Despite these developments, individuals with ASPD remain frequently excluded from personality disorder treatment programs (Sheehan et al., 2016) reflecting persistent clinical and systemic challenges.

Research on countertransference in ASPD and psychopathy, particularly on the variables that may influence it, can offer valuable guidance to clinicians in monitoring their emotional reactions, which is crucial for effectively navigating treatment challenges and establishing a strong therapeutic alliance (De Page et al., 2021). In addition, this line of research may provide useful indications for the development of therapeutic interventions for individuals with ASPD/psychopathy across both forensic and non-forensic settings.

Based on this background, the current scoping review aims to explore clinicians’ emotional reactions when delivering clinical care to individuals diagnosed with ASPD and/or psychopathy. In this article “emotional response,” “emotional reaction,” and “countertransference” are used interchangeably to refer to this construct (Colli et al., 2014). A secondary aim is to map clinicians’ personal opinions about the management of these emotional reactions to establish and preserve a strong therapeutic relationship. Understanding such opinions is relevant as they may potentially influence clinical decision-making.

## 2 Methods

### 2.1 Materials and methods

We performed a scoping review following the PRISMA-ScR guidelines (Khalil et al., 2021; Tricco et al., 2018) and The Joanna Briggs Institute Manual for Evidence Synthesis (Peters et al., 2015, 2020) (*a priori* registered protocol: https://osf.io/eaquj), in line with previous scoping reviews (Fornaro et al., 2021; Lo Buglio et al., 2024). The PRISMA-ScR checklist is provided in Supplementary material S1. Protocol amendments are reported in Supplementary material S2.

### 2.2 Search strategy and inclusion criteria

Two of the authors, UB and AM, performed a preliminary search on PubMed and Web of Science to identify potentially relevant primary studies. A full search strategy was developed based on the identified keywords, titles, and abstracts (see Supplementary material S3). This strategy was applied to PubMed, Web of Science, and EBSCO/PsycINFO databases to find studies published from 1990 to December 6, 2023. After removing duplicates, UB and AM screened titles and abstracts of the articles, including those that met the inclusion criteria (see below). Then, the same authors independently screened the studies at title/abstract and full-text levels. A third author (TB) was contacted to solve potential disagreements over the course of the screening process. Widely used research websites (e.g., ResearchGate) and the references of the retrieved articles, were also searched for additional reports.

We included studies that met the following criteria: (i) included mental health professionals providing clinical care to individuals with ASPD or psychopathy diagnosed via standardized and validated instruments (“population”); (ii) focused on any cognitive, emotional and behavioral responses of clinicians crucial for understanding their countertransference dynamics (“concept”); (iii) conducted in any context or location (“context”); (iv) used any type of primary study design (e.g., cross-sectional studies, randomized controlled trials, and cohort studies) (“type of study”) including gray literature (e.g., proceedings and dissertations); and (v) written in English. Beyond quantitative studies, we also included qualitative studies as they emphasize the participants’ own words, providing insights into their perspectives on complex phenomena.

### 2.3 Data extraction and presentation of findings

We extracted the following data from the retrieved studies: (a) demographic characteristics (e.g., country, mean age, gender, education and professional status of participants) and study design; (b) study aims and main findings; (c) instruments employed to assess ASPD or psychopathy and tools used to assess clinicians’ reactions toward individuals with ASPD or psychopathy; (d) details about clinical interventions (i.e., setting, therapeutic process, theoretical orientation and professional experience of the therapist); (e) relevant considerations regarding the clinical use of countertransference and possible intervention strategies; (f) authors’ suggestions for future research.

Data were extracted and organized in a dataset, which was updated throughout the study. A table was created to display the main characteristics of the included studies. Finally, to highlight and connect the key findings in this field, we conducted a narrative synthesis, organized by specific themes.

## 3 Results

**Figure 1:**
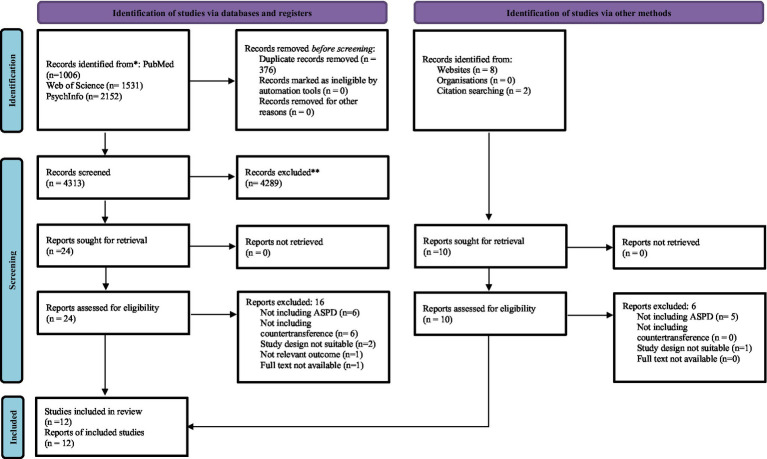
The PRISMA-ScR flow diagram of the literature search and the selection process.

Overall, 4,698 records were identified, 376 of which were duplicates. After excluding 4,313 records based on title/abstract screening and 24 after full-text review (detailed in Supplementary material S4), 12 studies were included in the current scoping review (Aerts et al., 2023; Cavalera et al., 2021; Colli et al., 2014; Di Virgilio et al., 2021; Gazzillo et al., 2015; Morken et al., 2022; Schwartz et al., 2007; Tanzilli et al., 2016, 2018, 2020; Van Dam et al., 2022; Warner and Keenan, 2022) (see Figure 1 and Table 1).

**Table 1:** Characteristics of the included studies.

Author and year	Study nationality	Study design	Study aims	Outcome	Setting	Patient characteristics	Psychotherapists characteristics	Treatment type	*N*	Measures	Main findings
Aerts et al. (2023)	The Netherlands	Qualitative	To gain insights into therapeutic aspects and processes in the TA when treating individuals with ASPD.	TA	Clinical and forensic	Mean Age: n/a Sex F/M: n/a Diagnosis: ASPD	Mixed (i.e., mental health psychologists, psychotherapists, clinical psychologists, psychiatrists) Mean Age: 45.6 Sex F/M: *F* = 11; M = 4 Theoretical orientation: not provided Main age of experience: 19.3	n/a	15	Semi-structured interviews	Respecting *patient’s needs, regulating interpersonal dynamics,* adopting a *connective attitude* and building the necessary *connective skills,* following the *treatment process* and striving for *collaborative and tailored goal*s are necessary to build TA with individuals with ASPD. Clinicians report that without having a strong TA, they risk losing the collaboration with the patient at any moment, leading to failure in treatment.
Cavalera et al. (2021)	Italy	Cross-sectional	To investigate the direct relationship between patients’ symptoms severity and therapist emotional response.	TR	Clinical	Mean Age: 28.7 Range: 18–48 Sex F/M: *F* = 26; M = 17 Diagnosis: PDs	Psychotherapists Mean Age: n/a Sex F/M: n/a Theoretical orientation: n/a Main age of experience: 8.93	n/a	19	TRQ, SWAP-200, SCL-90R	Antisocial factors were correlated with hostile/angry and overwhelmed/disorganized clinicians’ emotional responses, even when controlled for symptom severity, treatment duration, age and level of education of the patient and therapist experience.
Colli et al. (2014)	Italy	Cross-sectional	To examine the relationship between therapists’ emotional responses and individuals’ personality disorders and level of psycho-logical functioning.	TR	Clinical	Mean Age: 34 Range: 29.5–38.5 Sex F/M: *F* = 118; M = 85 Diagnosis: PDs	Mixed (i.e., psychotherapists, psychiatrists) Mean Age: 43 Sex F/M: *F* = 111 M = 92 Theoretical orientation:p sychodynamic = 103; cognitive-behavioral = 100 Main age of experience: 10	n/a	203	TRQ, SWAP-200	ASPD was correlated with criticized/mistreated response. Clinicians tend to feel mistreated, criticized, or repulsed and can experience an intense anger and irritation working with individuals with ASPD regardless of symptom severity.
Di Virgilio et al. (2021)	Belgium	Cross-sectional	Investigate patient symptomatology, inpatient violence, and antisocial/psychopathic personality traits impact CT	TR	Forensic	Mean Age: n/a Sex F/M: n/a Diagnosis: schizophrenia, not otherwise specified psychotic disorder, schizoaffective disorder. PDs, ID, SUD	Mixed (i.e., psychiatrists, psychologists, nurses, educators, caregivers) Mean Age: n/a Sex F/M: n/a Theoretical orientation: n/a Mean age of experience: n/a	n/a	163	TRQ, CAPP-IRS, PANSS, OAS, SOS	Criticized/Mistreated and disengaged TRQ scales, had a significantly stronger association with psychopathic traits. For psychologists/psychiatrists, psychopathic traits were negatively associated with parental/protective & positive/satisfying CT. For nurses, no CAPP-IRS domain appeared to have a significantly stronger association with CT; conversely, for psychologists/psychiatrists, the CAPP-IRS Self domain had the strongest association with CT.
Gazzillo et al. (2015)	Italy	Cross-sectional	to explore the relationships between individuals’ levels of personality organization and the specific personality disorders/patterns of the PDM P Axis with the emotional responses experienced by the therapists working with these individuals.	TR	Clinical	Mean Age: 36.5 Range: 17–75 Sex F/M: *F* = 82; M = 67 Diagnosis: PDs	Psychotherapists Mean Age: n/a Sex F/M: *F* = 87 M = 61 prefer not the answer = 1 Theoretical orientation: dynamic: 61; eclectic, but mainly dynamic: 48; an eclectic, but mainly biological: 20; cognitive– behavioral: 15; other: 4 Main age of experience: n/a	n/a	149	TRQ, PDP, PDC	An overwhelmed response was correlated with psychopathic patterns, which remained consistent even when overall level of personality organization was controlled
Morken et al. (2022)	Norway	Qualitative	Explore therapist experiences and therapist wellbeing in MBT ASPD	TR	Clinical	Mean age: 37 Range: 26–49 Sex F/M: n/a Diagnosis: ASPD	Mixed (i.e., Psychotherapists, social worker) Mean age: 42.4 Sex F/M: n/a Theoretical Orientation: MBT Main age of experience: T1:10, T2:18, T3:1, P1:14, P2: 7	MBT	5	Focus group discussion transcript analysis	By better understanding individuals with ASPD and their interactions with peers in group therapy, therapists experienced fewer negative preconceptions and greater confidence in their clear and concise roles. Maintaining boundaries and clear expectations, along with a non-judgmental stance, were essential strategies in MBT for ASPD. It is crucial to monitor countertransference and changes in therapists’ psychological functioning, with supportive measures in place to manage these issues. The MBT-ASPD group dynamic, characterized by high tempo and mocking humor, both excites and exhausts therapists.
Schwartz et al. (2007)	USA	Cross-sectional	To investigate psychotherapists’ CT reactions toward clients with ASPD and Schizophrenia	TR	Clinical	Mean age: n/a Sex F/M: n/a Diagnosis: ASPD or Schizophrenia	Graduate level students in psychotherapy programs (i.e., master’s and doctoral programs) Mean age: 33.03 Sex F/M: *F* = 55 M = 18 Theoretical orientation: CBT, solution-focused perspective, systemic-perspective, cognitive perspective, humanistic perspective, behavioral perspective. Main age of experience: 89.0% having fewer than 3 years of experience, and 57.5% having completed three or fewer years of graduate-level training	n/a	73	IMI, demographic questionnaire, client videotapes	Participants displayed significantly stronger feelings of being dominated by individuals with ASPD, indicating that they felt exploited, manipulated, and talked down to with individuals with ASPD diagnosis
Tanzilli et al. (2016)	Italy	Cross-sectional	To assess the factor structure, reliability, and validity of the TRQ	TR	Clinical	Mean Age: 40 Range: 25–66 Sex F/M: *F* = 174 M = 158 Diagnosis: DSM–IV–TR axis I, axis II diagnosis, ED, GAD, dysthymic disorder, Panic Disorder, SUD, PDs	Mixed (i.e., Psychiatrists, clinical psychologists) Mean Age: 47 years Sex F/M: *F* = 180 M = 152 Theoretical orientation: psychodynamis and cognitive behavioral Main age of experience: 10 years	Cognitive-behavioral or Psychodynamic psychotherapy	332	Demographic questionnaire, TRQ, SWAP-200, SCL-90R	SWAP–200 ASPD scale was positively associated with hostile/angry, criticized/devaluated therapist responses.
Tanzilli et al. (2018)	Italy	Cross-sectional	To investigate the associations among the scales of two different SWAP-200 FFM models, explore whether specific scales of the two models of SWAP-200 FFM traits were significantly associated with distinct SWAP dimensional scales of personality, and to examine the factor structure, reliability, and validity of the TRQ	TR	Clinical	Mean Age: 35.48 Range: 19–65 Sex F/M: *F* = 97 M = 69 Diagnosis: DSM–IV–TR axis I, axis II diagnosis, ED, GAD, dysthymic disorder, Panic Disorder, SUD, PDs	Mixed (i.e., Psychiatrist, clinical psychologists) Mean Age: 44.48 years Sex F/M: *F* = 95 M = 71 Theoretical Orientation: psychodynamic and cognitive-behavioral Main Age of Experience: 9.97 years	Cognitive-behavioral/ psychodynamic psychotherapy	166	Demographic questionnaire, SW-FFM, MLC-FFM, SWAP-200	The psychopathy personality traits positively correlated with the criticized/devaluated, overwhelmed/disorganized, hostile/angry and helpless/inadequate CT factors and negatively correlated with positive/satisfying and parental/protective CT factors.
Tanzilli et al. (2020)	Italy	Cross-sectional	To assess the TRQ-A factor structure, to investigate the associations between SWAP-II-A and in TRQ-A scales	TR	Clinical	Mean age: 16 Range: 13–18 Diagnosis: Dysthymic disorder, GAD, Panic disorder, Eating disorder, ADHD, Conduct disorder, SUD, ODD, PDs	Mixed (i.e., psychologists, psychiatrists) Mean age: 45 *F* = 121 M = 71 Theoretical Orientation: Psychodynamic & Cognitive Behavioral Mean age of Experience: 12.7	Cognitive-behavioral or Psychodynamic psychotherapy	192	SWAP-II-A; TRQ-A	The antisocial personality disorder scale was positively related to disengaged/hopeless CT and negatively associated with the warm/attuned therapist response. Correlations ranged from 0.15 to 0.31, indicating relatively small effect sizes.
Van Dam et al. (2022)	Netherlands	Cross-sectional	To investigate factors influencing clinicians’ willingness to work with individuals with ASPD	TR	Clinical and forensic	Mean age: n/a Sex F/M: n/a Diagnosis: ASPD	Mixed (i.e., psychiatrists, psychiatric nurses, psychologists, social workers) Mean age Psychiatrist: 42 F = 4 M = 6 Main Age of Experience: 12.7 Mean age Psychologist: 37.4 F = 56 M = 18 Main Age of Experience: 11.9 Mean age Social Worker: 51.1 F = 9 M = 5 Main Age of Experience: 19.0 Mean age Psychiatric Nurse: 40.8 Main Age of Experience: 17.7 F = 19 M = 13 Theoretical Orientation: n/a	Psychological treatment	130	TPBaspdT; FWC-30	The study confirms the limited motivation to work with ASPD patients in regular mental health care settings. The motivation to work with individuals with ASPD was higher in forensic settings, associated with more positive attitude, higher perceived social norm, more perceived behavior control, less negative emotions and more positive emotions in forensic mental health compared to regular mental health service. However even in forensic mental health care, one third of the clinicians are not motivated or ambivalent about providing mental help to this group of patients The level of motivation is not explained by experience, education on cluster B personality disorders and having experienced verbal and/or physical violence in clinical practice. However negative emotions were related to less motivation, yet positive emotions appeared to be more strongly associated with the motivation to provide treatment to ASPD patients.
Warner and Keenan (2022)	England	Qualitative	To investigate the experiences and challenges of clinicians delivering MBT for individuals with ASPD.	TR	Forensic	Mean age: n/a Sex F/M: M Diagnosis: ASPD	Mixed (i.e., Clinical Psychologist, Group therapist) Mean age: n/a Mean age of Experience: n/a F = 5 M = 1 Theoretical Orientation: n/a	MBT	6	semi-structured interviews	Clinicians felt vulnerable and inadequate working with individuals with ASPD. They felt responsible for the insufficient service infrastructure which led to breaching their own time boundaries to support their clients, risking burnout and emotional exhaustion. Their self-sacrifice was also driven by a desire to protect the competent and comprehensive professional identity which they perceived to be at risk.

ADHD, attention-deficit/hyperactivity disorder; ASPD, Antisocial Personality Disorder; CAPP-IRS, the Comprehensive Assessment of Psychopathic Personality-Institutional Rating Scale; ED, eating disorder; FFM, Five factor model; GAD, generalized anxiety disorder; FWC-30, Feeling Word Checklist; IMI-ID, Intellectual Disability, Impact Message Inventory; n/a, not availale; MBT, Mentalization-Based Treatment; MLC-FFM, McCrae, Löckenhoff, and Costa-Five factor model; OAS, Overt Aggression Scale; ODD, oppositional defiant disorder; PANSS, The Positive and Negative Syndrome Scale; PDs, Personality Disorders; PDC, Psychodiagnostic chart; PDP, Psychodynamic Diagnostic Prototypes; Q, qualitative study design; SCL-90-R, symptoms checklist 90 revised; SOS, START Outcome Scale; SUD, substance use disorder; SWAP-200, Schedler and Westen Assessment Procedure; SWAP-II-A, Shedler-Westen assessment procedure for adolescents version II; SW-FFM, Shedler and Westen-Five factor model; TA, Therapeutic Alliance; TPBaspdT, Theory of Planned Behavior Questionnaire for ASPD Treatment; TR, Therapist Response; TRQ, Therapist Response Questionnaire; TRQ-A, Therapist response questionnaire for adolescents.

Most studies were conducted in Europe (*n* = 11), only one (Schwartz et al., 2007) was conducted in North America, and they spanned from 2000 to 2023. Nine studies employed cross-sectional designs, and three employed qualitative designs conducted in forensic (*n* = 3) or non-forensic (*n* = 9) mental health settings.

The number of clinicians ranged from 4 (Morken et al., 2022) to 203 (Colli et al., 2014) across studies, with various professional backgrounds including clinical psychologists, psychiatrists, psychotherapists, social workers, educators, and psychiatric nurses. Clinicians’ experience ranged from graduate students in psychotherapy programs (Schwartz et al., 2007) to professionals with over 19 years of experience (Aerts et al., 2023). Measures for assessing clinicians’ emotional responses included the Therapist Response Questionnaire (TRQ) (Betan et al., 2005; Colli et al., 2014) and the Therapist Response Questionnaire for Adolescents (TRQ-A) (Satir et al., 2009), as the most used, the Impact Message Inventory (IMI) (Kiesler, 1987), the Feeling Word Checklist (FWC-30) (Whyte et al., 1982), and the Theory of planned behavior questionnaire for ASPD treatment (TPBaspdT) (Ajzen, 2013). Further, three studies (Aerts et al., 2023; Morken et al., 2022; Warner and Keenan, 2022) employed a qualitative research methodology, where in-depth interviews or focus groups were explored through thematic analysis (Aerts et al., 2023; Braun and Clarke, 2006), Interpretative Phenomenological Analysis (IPA) (Smith et al., 2008; Warner and Keenan, 2022), or an autoethnographic self-reflective approach (Morken et al., 2022; Råbu et al., 2021).

Tools for assessing ASPD included the Shedler-Westen Assessment Procedure-200 (SWAP-200) (Westen and Shedler, 1999a, 1999b) and the Shedler-Westen Assessment Procedure for Adolescents (SWAP-II-A) (Westen et al., 2003) as the most used, the Psychodiagnostics chart (PDC) (Gordon and Bornstein, 2012), and the SCID-5-PD (First et al., 2016). Three studies focused on psychopathy (Di Virgilio et al., 2021; Gazzillo et al., 2015; Tanzilli et al., 2018) assessed by the Comprehensive Assessment of Psychopathic Personality-Institutional Rating Scale (CAPP-IRS) (Cooke et al., 2017), or the Q-factor model of the SWAP-200 (Shedler and Westen, 2004).

The patients to whom the recruited clinicians provided clinical care were adults (Cavalera et al., 2021; Colli et al., 2014; Tanzilli et al., 2016, 2018), adolescents (Tanzilli et al., 2020), or adolescents and adults (Gazzillo et al., 2015). One study explored a sample of individuals with psychotic disorders and comorbid ASPD (Di Virgilio et al., 2021). Patients’ age was not provided in five studies (Aerts et al., 2023; Di Virgilio et al., 2021; Schwartz et al., 2007; Van Dam et al., 2022; Warner and Keenan, 2022), while in one case the sample consisted only of male subjects (Warner and Keenan, 2022).

## 4 Common emotional responses

Most studies (Cavalera et al., 2021; Colli et al., 2014; Di Virgilio et al., 2021; Gazzillo et al., 2015; Schwartz et al., 2007; Tanzilli et al., 2016, 2018, 2020; Van Dam et al., 2022) directly explored the emotional responses of clinicians working with individuals with ASPD and/or psychopathy through validated instruments, while three studies (Aerts et al., 2023; Morken et al., 2022; Warner and Keenan, 2022) applied a qualitative research methodology to interviews or focus groups.

In Schwartz et al. (2007), therapists experienced fewer positive emotions (i.e., being liked and welcomed and being in charge) while working with individuals with ASPD compared to those with schizophrenia. In the context of a qualitative interview, Morken et al. (2022) showed that four experienced therapists typically felt nervous before sessions and feared aggressive outbursts or conflicts in mentalization-based treatment (Bateman and Fonagy, 2008) group sessions. They also found that while some therapists became more “badass” and less afraid, others felt fearful and hypervigilant on the external environment and patient behaviors (Morken et al., 2022). Schwartz et al. (2007) also noted that therapists perceived individuals with ASPD as evoking a sense of concerns about being exploited, harassed, demeaned, or controlled.

Notably, individuals with ASPD often exhibit little to no contact with vulnerable emotions and display playful or oppositional behaviors (Morken et al., 2022). The authors of the study reported that these behaviors led them (i.e., therapists and one social worker) to feel emotionally detached or entertained, hindering effective interventions. Some therapists also expressed concerns about developing a tolerance for aggressivity and violence, recognizing that this could hinder their ability to provide effective treatment. To address this, clinicians emphasized the importance of supervision (Morken et al., 2022).

Seven studies (Cavalera et al., 2021; Colli et al., 2014; Di Virgilio et al., 2021; Gazzillo et al., 2015; Tanzilli et al., 2016, 2018, 2020) investigated therapists’ countertransference using the TRQ (Betan et al., 2005; Tanzilli et al., 2016) when working with individuals with personality disorders. Findings suggest that similar countertransference patterns were associated with ASPD in a consistent and predictable way, beyond the theoretical orientations of the clinicians involved in the studies.

ASPD was related to “hostile/angry” (Cavalera et al., 2021; Tanzilli et al., 2016) and “criticized/devalued” (Tanzilli et al., 2016) emotional responses, which include feelings of anger and irritation, and feelings of being devalued, criticized or repulsed, used or manipulated and pushed to set firm limits in the clinical setting, respectively. Similarly, Colli et al. (2014) found a significant correlation between ASPD and “criticized/mistreated” emotional response (i.e., a TRQ subscale subsequently split into the above-mentioned factors in a later factorial structure), with this response pattern remaining consistent regardless of symptom severity, therapeutic approach, and clinician characteristics. Additionally, Cavalera et al. (2021) found that ASPD was predictive of the overwhelmed/disorganized (i.e., urge to avoid the patient, combined with strong negative emotions like repulsion and resentment) response, which did not emerge in other studies. These results persisted even when controlling for symptom severity, duration of treatment, patient age and educational level, and therapist experience (Cavalera et al., 2021). By recruiting clinicians working with adolescent patients, one study (Tanzilli et al., 2020) found antisocial traits and disorders to be correlated to clinicians’ “disengaged/hopeless” emotional response, characterized by feelings of disconnection and pessimism about the therapeutic process.

Regarding clinicians working with patients with psychopathy, “overwhelmed/disorganized” response was correlated with psychopathy personality traits in two studies (Gazzillo et al., 2015; Tanzilli et al., 2018). In Gazzillo et al. (2015) this finding remained even after controlling for the overall level of individuals’ personality organization (i.e., a spectrum of personality functioning from healthy, through neurotic and borderline, to psychotic levels) (Lingiardi and McWilliams, 2015a; McWilliams and Lingiardi, 2017). Furthermore, psychopathic traits were significantly associated with “hostile/angry” and “criticized/devalued” response in Tanzilli et al. (2018), and with “criticized/mistreated” response in Di Virgilio et al. (2021). Both studies found that psychopathic personality traits were negatively associated with positive/satisfying (i.e., feeling a positive working alliance and connection with the individual in therapy) and parental/protective (i.e., a desire to protect and nurture the individual in therapy in a parental manner) responses (Di Virgilio et al., 2021; Tanzilli et al., 2018).

Additionally, Tanzilli et al. (2018) found positive correlations between psychopathy and “helpless/inadequate” response, characterized by feelings of anxiety, incompetency, and inadequacy. On the other hand, Di Virgilio et al. (2021) found that psychopathic traits were significantly associated with disengaged reactions, which involve feeling distracted, withdrawn, annoyed, or bored in sessions. In Di Virgilio et al. (2021) the psychopathy “self” domain (i.e., encompassing self-centered traits, a sense of uniqueness, entitlement, and invulnerability) had the strongest association with these response patterns.

Overall, the studies employing the TRQ highlighted that clinicians frequently experienced strong negative emotions toward individuals with psychopathy and ASPD in therapy, including feeling devalued, hostility, anger, overwhelmed or experiencing repulsion. Also, psychopathy personality patterns overall reduce the protective and positive attitude of clinicians.

## 5 Willingness to deliver clinical care

One study (Van Dam et al., 2022) addressed motivation in working with individuals with ASPD in both forensic and regular mental health care settings.

In traditional mental health care settings, approximately 60% of clinicians (i.e., psychiatrists, psychologists, social workers, and psychiatry nurses) were found to lack motivation to work with individuals diagnosed with ASPD, with only 12% expressing willingness to engage them. In contrast, forensic mental health care settings, where ASPD diagnoses are more prevalent, exhibited a higher proportion of motivated clinicians (65%) (Van Dam et al., 2022). The most significant factor influencing the provision of psychological treatment was the clinician’s perception of having knowledge and skills to provide clinical interventions leading to “good results” (Ajzen, 2013). In contrast, experience in treating individuals with ASPD did not significantly explain willingness. Surprisingly, having experienced verbal and/or physical violence in clinical practice did not affect motivation. Based on these findings, the authors suggested that clinicians working with individuals with ASPD may benefit from training and supervision focused on enhancing their feelings of control and competence.

## 6 Emotional responses related to misogynistic behaviors

One study (Morken et al., 2022) discussed the potential influence of gender dynamics in the context of group therapy with ASPD individuals. Clinicians (i.e., therapists and one social worker) reported that individuals with ASPD often engage in unpredictable and inappropriate behaviors, without considering the potential discomfort they might cause to other individuals. These behaviors include making derogatory statements about women, asking intrusive questions about personal and sexual topics, or mocking the therapist in both group and individual therapy sessions. Notably, female therapists reported difficulties in managing these misogynistic behaviors while maintaining a mentalizing stance (i.e., the capability to reflect on own and others mental states and adopting this ability in the context of social interactions) (Bateman and Fonagy, 2008). On such occasions, female therapists reported feeling overwhelmed, losing their ability to think clearly, and struggling with how to react while safeguarding professional boundaries. Moreover, therapists reported that when they express their discomfort and address the inappropriateness of such dialogues, individuals with ASPD either immediately cease and retract their statements, or claim they were joking and did not mean them literally (Morken et al., 2022).

## 7 Opinions about clinical management and therapeutic alliance

Two studies (Aerts et al., 2023; Morken et al., 2022) interviewed clinicians to explore their experience when working with ASPD individuals, providing results on clinicians’ opinions on therapeutic relationship and working alliance management. According to the therapists in the study of Aerts et al. (2023), developing a trustful patient-clinician relationship characterized by a secure and non-judgmental space is crucial for establishing a working alliance. One clinician expressed: “They have been neglected so often that being heard, being taken seriously, the recognition of what they think and feel, that they matter to others, the autonomy and the safety, are very important aspects” (Aerts et al., 2023). Accordingly, the authors emphasized that individuals with ASPD highly appreciate being respected and recognized as unique individuals with their own qualities and talents (Aerts et al., 2023).

However, building a therapeutic alliance may be challenging, due to the increased challenges in setting boundaries compared to individuals without ASPD. Aerts et al. (2023) recommended to adopt a firm and flexible approach to tailor clinical intervention to each patient’s needs, also avoiding an authoritarian attitude, as individuals with ASPD may be sensitive to such a behavior and may respond with resistance. In contrast, in the context of group therapies, the clinicians (i.e., therapists and one social worker) in the study of Morken et al. (2022) highlighted the importance of maintaining an authoritative therapeutic stance (i.e., being capable to keep individuals on the therapy tasks) and setting clearly defined boundaries and expectations, along with a non-judgmental stance (Morken et al., 2022).

## 8 Discussion

This scoping review mapped the existing literature on mental health professionals’ emotional reactions when working with individuals diagnosed with ASPD or psychopathy. When confronted to these subjects, clinicians tend to experience complex emotional responses and ambivalent reactions, which can be challenging to recognize and address. Such reactions need to be identified and managed, as they may have a significant impact not only on the treatment process, but also on the clinician’s willingness to work with these patients. Finally, in line with previous research on this topic (Pallagrosi et al., 2016; Picardi et al., 2017), our findings support the diagnostic value of countertransference in therapy with individuals with ASPD or psychopathy.

Mental health professionals working with individuals diagnosed with ASPD or psychopathy seem to be exposed to a variety of different emotional reactions. First, psychopathic personality traits have been negatively associated with positive/satisfying (i.e., feeling a connection with the person and experiencing a positive working alliance) and parental/protective (i.e., a disposition to protect and nurture the person in a parental manner) responses in mental health professionals (Di Virgilio et al., 2021; Tanzilli et al., 2018). Second, clinicians experience complex negative emotional reactions which seem to involve an active, “antagonized” side, including feelings of anger for being exploited, deceived, criticized, harassed, degraded or controlled (Cavalera et al., 2021; Colli et al., 2014; Di Virgilio et al., 2021; Schwartz et al., 2007; Tanzilli et al., 2016, 2018), and a passive, “overwhelmed” side, including concern about personal safety (Morken et al., 2022), a sense of disorganization and being overwhelmed (Cavalera et al., 2021; Gazzillo et al., 2015; Tanzilli et al., 2018), and feelings of detachment, inadequacy, helplessness, and lack of motivation (Di Virgilio et al., 2021; Tanzilli et al., 2018, 2020). A recent review showed that, in individual psychotherapy settings, antisocial personality traits are positively associated with negative feelings of being mistreated, criticized, and devalued, as well as with feelings of annoyance and anger (Stefana et al., 2020).

The therapeutic relationship with these individuals is particularly challenging due to the complex and non-linear emotional reactions they tend to elicit in the clinician. Mental health professionals may find themselves either entertained by the easy-going and seductive attitude of these individuals or concerned about developing a tolerance for aggressivity and violence (Morken et al., 2022). This is in line with longstanding psychoanalytic literature, which suggests that countertransference responses to subjects with ASPD are often marked by negative feelings ranging from devaluation to overt moral condemnation (Symington, 2001), as well as by feelings of admiration or envy, which may lead to an illusion of alliance (Gerstley et al., 1989) or to a denial of the patient’s harmfulness (Lion, 2001).

Notably, most of these feelings seem to be independent of different variables, such as gender, age, profession, clinical experience and theoretical orientation of the mental health professional (Cavalera et al., 2021; Colli et al., 2014; Tanzilli et al., 2020; Van Dam et al., 2022) In contrast, emotional reactions seem to be affected by the patient’s overall level of psychological functioning, since those with higher levels of functioning tend to evoke more positive reactions (Colli et al., 2014), while less organized patients tend to elicit feelings of helplessness, overwhelm, and a sense of inadequacy in establishing a good therapeutic alliance (Gazzillo et al., 2015). These findings align with the literature on individual psychotherapy settings, which suggests that working with emotionally dysregulated patients is associated with therapist’s feelings of anxiety and incompetence (Stefana et al., 2020).

When compared to subjects affected by schizophrenia, those with ASPD are more likely to be rated by psychotherapists as higher on the Dominance scale of the Impact Message Inventory, which reflects perceptions of being exploited, manipulated, bossed around, talked down to, and dominated in interpersonal contexts (Schwartz et al., 2007). Moreover, among psychologists and psychiatrists, an association was found between negative countertransference reactions and the Self domain of the Comprehensive Assessment of Psychopathic Personality-Institutional Rating Scale (CAPP-IRS), which encompasses self-centered traits, a sense of uniqueness, entitlement, and invulnerability (Di Virgilio et al., 2021). Similarly, narcissistic personality disorder is also positively associated with hostile/angry, criticized/devalued, helpless/inadequate, and disengaged countertransference (Tanzilli et al., 2018).

The only study on female therapists’ perspectives when working with patients with ASPD (Morken et al., 2022) showed that the misogynistic behavior of male ASPD patients may hinder the therapist’s ability to maintain a mentalizing stance. This might also be related to the tendency of these patients to seek dominant roles across a wide range of interpersonal contexts. Although limited research has explored the impact of gender on the clinician’s emotional reactions, preliminary evidence indicates that female clinicians may feel overwhelmed when working with male patients. In contrast, male clinicians tend to feel angrier with male patients and more receptive toward female patients diagnosed with ASPD (de Vogel and Louppen, 2016). It is crucial to understand the role of unconscious sexualization in the context of the therapeutic relationship, since such patients may use playful and charming attitudes to effectively “seduce” the clinician, who may fail to understand their behaviors.

Longstanding theoretical contributions conceptualize antisocial behaviors as a primitive variants of the narcissistic personality continuum, ranging from subclinical antisocial behaviors through malignant narcissism to full-blown ASPD or psychopathy (Kernberg, 1984; Meloy, 1988). The severity along this continuum is shaped by the structure of the narcissistic self and by the individual’s capacity to form meaningful interpersonal relationships and to experience guilt or remorse. At one end of the spectrum, ASPD and psychopathy are characterized not only by a pathologically grandiose Self but also by the inability to authentically depend on others (Kernberg, 2020). The development of ASPD and psychopathy may be associated with a profound detachment from all relationships and affective experiences, often accompanied by sadistic attempts to connect with others through power dynamics (Meloy, 1988). These processes may impair the development of meaningful relationships with others and prevent the development of a strong therapeutic alliance.

Most common emotional responses to ASPD/psychopathic patients are relatively consistent and independent of several variables, such as the severity of symptoms and the theoretical orientation of the therapist. When working with individuals with ASPD or psychopathy, it may be crucial to identify emotional reactions such as anger, emotional overwhelm, or detachment, as these may reflect the sadistic relational patterns rooted in the patient’s narcissistic core, and help guide the planning of tailored intervention strategies (Kernberg, 2020; Meloy, 1988).

Negative countertransference patterns influence the clinician’s willingness to work with ASPD patients, especially in regular mental health care settings (Van Dam et al., 2022). These emotional reactions, especially when associated with prejudices, may eventually result in therapeutic nihilism (Meloy and Yakeley, 2014), potentially leading to the exclusion of ASPD individuals from treatment programs (Sheehan et al., 2016; Van Dam et al., 2022; van den Bosch et al., 2018). Conversely, according to our results, professionals working in forensic mental health care settings, where ASPD diagnosis is more prevalent, seem to be more motivated to treat these individuals (Van Dam et al., 2022). In these contexts, clinicians often lack the option to reject patients—who are typically mandated to treatment by the court—fosters continuous exposure, which over time may reduce initial resistance and trepidation (Seaward et al., 2021). Importantly, this difference does not solely reflect clinical experience, but also a difference in mindset: forensic clinicians, required by institutional duty to treat ASPD individuals, often learn to engage with them more intentionally and strategically, whereas non-forensic clinicians may feel freer to avoid these cases, perceiving them as untreatable (Hachtel et al., 2019). This highlights the relevance of training and structured support. Developing clear treatment models for ASPD, combined with ongoing supervision, can enhance clinicians’ sense of competence and reduce the emotional burden associated with these patients (Flaaten et al., 2024).

Interestingly, in this clinical population, the level of professional experience alone does not seem to be a protective factor against negative emotional reactions (Cavalera et al., 2021; Morken et al., 2022). In contrast, preliminary evidence suggests that the clinician’s self-perception as a competent and skillful professional providing effective clinical interventions is the most important factor influencing the willingness to provide psychological treatment to ASPD/psychopathic patients (Van Dam et al., 2022). This is in line with the Theory of Planned Behavior (Ajzen, 2013), which posits that attitude is the most relevant factor influencing one’s disposition toward treatment, as it can predict the motivation to provide care. Based on this framework, fostering a more positive attitude may require clinicians to adopt the belief that: (a) effective and beneficial treatment options are available for individuals with ASPD—that is, improvement is possible within clinical care—and (b) working with these individuals can be a stimulating and intellectually challenging task (Van Dam et al., 2022).

Emotional reactions may also include feelings of interest or engagement on the part of the clinician, highlighting the complexity and variability of emotional reactions when working with this population. However, further research is needed to explore the role of positive emotions toward individuals with ASPD or psychopathy, and their potential impact on therapeutic outcomes. A recent study (Flaaten et al., 2024) identified four major obstacles that contribute to the therapeutic pessimism toward this clinical group, namely confusion surrounding psychopathy/ASPD, treatment-rejecting behaviors, refusing ASPD patients from treatment, and inadequate management of countertransference. The authors of that study suggested that a manualized and structured intervention, such as the mentalization-based treatment for ASPD (MBT-ASPD), may show promise for addressing these challenges (Flaaten et al., 2024). This was recently supported by a multicenter trial on MBT, which reduced individual self-defeating and interpersonally violent core features in ASPD male patients (Fonagy et al., 2025).

The current scoping review provides a comprehensive overview of the specific and relatively underexplored topic of countertransference in the treatment of ASPD/psychopathic patients. We highlighted areas that require further investigation, such as the role of staff gender and the difference in the therapist’s emotional reactions between ASPD and psychopathy, paving the way for future studies. Moreover, we incorporated both qualitative and quantitative evidence.

However, there are also some limitations that should be acknowledged. First, cohort studies and randomized controlled trials exploring therapists’ emotional responses toward individuals with ASPD were not available. Second, the heterogeneity of measures adopted limits the comparisons across findings. Third, we did not retrieve studies on the therapists’ personalities, vulnerabilities, and unresolved issues (Tishby and Wiseman, 2022), all of which can impact countertransference reactions (Tanzilli et al., 2020). Fourth, in two studies, participants with antisocial traits or disorders were under the age of 18 (Gazzillo et al., 2015; Tanzilli et al., 2020); however, a growing body of evidence indicates that emerging patterns of personality pathology in adolescence are highly prevalent and persistent (Hamlat et al., 2020; Kongerslev et al., 2014; Tanzilli et al., 2024). Finally, we did not include studies on conduct disorder as a part of the “antisociality” developmental continuum.

Future research could focus on cross-cultural differences (Altuncu et al., 2023; Boldrini et al., 2024) and specific settings (Pontillo, et al., 2020)—such as forensic settings—and examine therapeutic approaches alongside clinicians’ emotional responses. Individual differences in emotional responses to patients with ASPD/psychopathy remain poorly understood and warrant closer investigation. Future studies may also consider countertransference as a ubiquitous and potentially useful phenomenon that should be investigated from a second-person perspective (Fuchs, 2010; Rocco et al., 2021), recognizing that meaning-making and insight are co-constructed within the therapeutic relationship (Tanzilli et al., 2020).

## Data Availability

The original contributions presented in the study are included in the article/Supplementary material further inquiries can be directed to the corresponding author.
