# Supplementary material for: Exploring mental health professionals’ emotional responses with individuals diagnosed with antisocial personality disorder or psychopathy: a scoping review
Source: Front Psychol. 2025 Jun 25;16:1501273. doi: 10.3389/fpsyg.2025.1501273 (PMC12239656; doi:10.3389/fpsyg.2025.1501273)
Supplement: Supplementary file 1 [file Data_Sheet_1.docx]

Supplementary materials to

**Exploring Mental Health Professionals' Emotional Responses with Individuals Diagnosed with Antisocial Personality Disorder or Psychopathy: A Scoping Review**

**Supplementary Materials S1**

Preferred Reporting Items for Systematic reviews and Meta-Analyses extension for Scoping Reviews (PRISMA-ScR) Checklist

| **SECTION** | **ITEM** | **PRISMA-ScR CHECKLIST ITEM** | **REPORTED ON PAGE #** |
| --- | --- | --- | --- |
| **TITLE** | | | |
| Title | 1 | Identify the report as a scoping review. | Title page |
| **ABSTRACT** | | | |
| Structured summary | 2 | Provide a structured summary that includes (as applicable): background, objectives, eligibility criteria, sources of evidence, charting methods, results, and conclusions that relate to the review questions and objectives. | Abstract |
| **INTRODUCTION** | | | |
| Rationale | 3 | Describe the rationale for the review in the context of what is already known. Explain why the review questions/objectives lend themselves to a scoping review approach. | Y |
| Objectives | 4 | Provide an explicit statement of the questions and objectives being addressed with reference to their key elements (e.g., population or participants, concepts, and context) or other relevant key elements used to conceptualize the review questions and/or objectives. | Y |
| **METHODS** | | | |
| Protocol and registration | 5 | Indicate whether a review protocol exists; state if and where it can be accessed (e.g., a Web address); and if available, provide registration information, including the registration number. | Y |
| Eligibility criteria | 6 | Specify characteristics of the sources of evidence used as eligibility criteria (e.g., years considered, language, and publication status), and provide a rationale. | Y |
| Information sources* | 7 | Describe all information sources in the search (e.g., databases with dates of coverage and contact with authors to identify additional sources), as well as the date the most recent search was executed. | Y |
| Search | 8 | Present the full electronic search strategy for at least 1 database, including any limits used, such that it could be repeated. | Supplementary  Materials |
| Selection of sources of evidence† | 9 | State the process for selecting sources of evidence (i.e., screening and eligibility) included in the scoping review. | Y |
| Data charting process‡ | 10 | Describe the methods of charting data from the included sources of evidence (e.g., calibrated forms or forms that have been tested by the team before their use, and whether data charting was done independently or in duplicate) and any processes for obtaining and confirming data from investigators. | Y |
| Data items | 11 | List and define all variables for which data were sought and any assumptions and simplifications made. | Y |
| Critical appraisal of individual sources of evidence§ | 12 | If done, provide a rationale for conducting a critical appraisal of included sources of evidence; describe the methods used and how this information was used in any data synthesis (if appropriate). | Not applicable |
| Synthesis of results | 13 | Describe the methods of handling and summarizing the data that were charted. | Y |
| **RESULTS** | | | |
| Selection of sources of evidence | 14 | Give numbers of sources of evidence screened, assessed for eligibility, and included in the review, with reasons for exclusions at each stage, ideally using a flow diagram. | Y |
| Characteristics of sources of evidence | 15 | For each source of evidence, present characteristics for which data were charted and provide the citations. | Y; Table 1 |
| Critical appraisal within sources of evidence | 16 | If done, present data on critical appraisal of included sources of evidence (see item 12). | Not applicable |
| Results of individual sources of evidence | 17 | For each included source of evidence, present the relevant data that were charted that relate to the review questions and objectives. | Y |
| Synthesis of results | 18 | Summarize and/or present the charting results as they relate to the review questions and objectives. | Y |
| **DISCUSSION** | | | |
| Summary of evidence | 19 | Summarize the main results (including an overview of concepts, themes, and types of evidence available), link to the review questions and objectives, and consider the relevance to key groups. | Y |
| Limitations | 20 | Discuss the limitations of the scoping review process. | Y |
| Conclusions | 21 | Provide a general interpretation of the results with respect to the review questions and objectives, as well as potential implications and/or next steps. | Y |
| **FUNDING** | | | |
| Funding | 22 | Describe sources of funding for the included sources of evidence, as well as sources of funding for the scoping review. Describe the role of the funders of the scoping review. | Y |

JBI = Joanna Briggs Institute; PRISMA-ScR = Preferred Reporting Items for Systematic reviews and Meta-Analyses extension for Scoping Reviews.

* Where *sources of evidence* (see second footnote) are compiled from, such as bibliographic databases, social media platforms, and Web sites.

† A more inclusive/heterogeneous term used to account for the different types of evidence or data sources (e.g., quantitative and/or qualitative research, expert opinion, and policy documents) that may be eligible in a scoping review as opposed to only studies. This is not to be confused with *information sources* (see first footnote).

‡ The frameworks by Arksey and O’Malley (6) and Levac and colleagues (7) and the JBI guidance (4, 5) refer to the process of data extraction in a scoping review as data charting*.*

§ The process of systematically examining research evidence to assess its validity, results, and relevance before using it to inform a decision. This term is used for items 12 and 19 instead of "risk of bias" (which is more applicable to systematic reviews of interventions) to include and acknowledge the various sources of evidence that may be used in a scoping review (e.g., quantitative and/or qualitative research, expert opinion, and policy document).

*From:* Tricco AC, Lillie E, Zarin W, O'Brien KK, Colquhoun H, Levac D, et al. PRISMA Extension for Scoping Reviews (PRISMAScR): Checklist and Explanation. Ann Intern Med. 2018;169:467–473. [doi: 10.7326/M18-0850](http://annals.org/aim/fullarticle/2700389/prisma-extension-scoping-reviews-prisma-scr-checklist-explanation).

**Supplementary Materials S2**

**Protocol amendements**

**Search Strategy:** ProQuest was initially listed as a database to be searched but was ultimately not included in the review process.

**Study Aim:** A secondary aim was introduced during the review to map clinicians' personal opinions on managing individuals with Antisocial Personality Disorder (ASPD) clinically, as we realize that it was clinically relevant.

**Supplementary Materials S3**

**Search strategy**

("Emotional Reactions" OR "Affect*" OR "Burnout" OR "Stress" OR "Compassion Fatigue") AND ("Clinicians" OR "Healthcare Providers" OR "Therapists" OR "Psychiatrists" OR "Nurses" OR "Healthcare Workers") AND ("Antisocial Personality Disorder" OR "ASPD" OR "Psychopath*" OR "Sociopath*" OR "Dissocial Personality Disorder" OR “Offender*")

**Supplementary Materials S4.** Excluded studies at full-text level with reasons

| **Author** | **Reason for exclusion at full-text level** |
| --- | --- |
| Awad., 1981 [(Awad, 1981)](https://www.zotero.org/google-docs/?tvEWK9) | Study design not suitable |
| Betan et al., 2005 [(Betan et al., 2005)](https://www.zotero.org/google-docs/?WMEbuG) | Not including ASPD |
| Carney., 1977 [(Carney, 1977)](https://www.zotero.org/google-docs/?67asnH) | Not including ASPD |
| Colli & Ferri., 2015 [(Colli & Ferri, 2015)](https://www.zotero.org/google-docs/?cCVA3p) | Study design not suitable |
| Crandal et al., 2015 [(Crandal et al., 2015)](https://www.zotero.org/google-docs/?ePS5b8) | Not including ASPD |
| De Page et al., 2021 [(De Page et al., 2021)](https://www.zotero.org/google-docs/?GOsVfb) | Not including ASPD |
| DeSorcy et al., 2020 [(DeSorcy et al., 2020)](https://www.zotero.org/google-docs/?dnH4yy) | Not including countertransference |
| Freestone et al., 2015 [(Freestone et al., 2015)](https://www.zotero.org/google-docs/?sR40oj) | Not including ASPD |
| Gerbrandij et al., 2022 [(Gerbrandij, 2022)](https://www.zotero.org/google-docs/?Cvu8LV) | Full text not available |
| Graham., 1980 [(Graham, 1980)](https://www.zotero.org/google-docs/?mQgmZd) | Not including ASPD |
| Kazdin et al., 2005 [(Kazdin et al., 2005)](https://www.zotero.org/google-docs/?mMFwia) | Not including countertransference |
| Kazdin et al., 2006 [(Kazdin et al., 2006)](https://www.zotero.org/google-docs/?6asjaB) | Not including countertransference |
| Knaus et al., 2016 [(Knaus et al., 2016)](https://www.zotero.org/google-docs/?KWqOYs) | Not including ASPD |
| Lingiardi et al., 2015 [(Lingiardi et al., 2015)](https://www.zotero.org/google-docs/?0fQD2x) | Not relevant outcome |
| Michaud et al., 2020 [(Michaud et al., 2020)](https://www.zotero.org/google-docs/?NvqTW8) | Not including ASPD |
| Polaschek et al., 2010 [(Polaschek & Ross, 2010)](https://www.zotero.org/google-docs/?HWyEzV) | Not including ASPD |
| Rossberg et al., 2007 [(Rossberg et al., 2007)](https://www.zotero.org/google-docs/?FstIxz) | Not including ASPD |
| Stefana et al., 2020 [(Stefana et al., 2020)](https://www.zotero.org/google-docs/?gJW5Ul) | Study design not suitable |
| Tanzilli et al., 2022 [(Tanzilli et al., 2022)](https://www.zotero.org/google-docs/?LXTGR8) | Not including ASPD |
| Thompson et al., 2014 [(Thompson et al., 2014)](https://www.zotero.org/google-docs/?xlrb4r) | Not including countertransference |
| Walton et al., 2018 [(Walton et al., 2018)](https://www.zotero.org/google-docs/?wYBFmZ) | Not including countertransference |
| TREATMENT OUTLINES FOR ANTISOCIAL PERSONALITY DISORDER [(“Treatment Outlines for Antisocial Personality Disorder,” 1991)](https://www.zotero.org/google-docs/?Fdcw7u) | Not including countertransference |

Exclusion reasons

1- Not including ASPD

2- Not including countertransference

3- Study design not suitable

4- Full text not available

**Supplementary references**

[Awad, G. A. (1981). The Early Phase of Psychotherapy with Antisocial Early Adolescents ^*^. *The Canadian Journal of Psychiatry*, *26*(1), 38–42. https://doi.org/10.1177/070674378102600108](https://www.zotero.org/google-docs/?vKWcPj)

[Betan, E., Heim, A. K., Zittel Conklin, C., & Westen, D. (2005). Countertransference Phenomena and Personality Pathology in Clinical Practice: An Empirical Investigation. *American Journal of Psychiatry*, *162*(5), 890–898. https://doi.org/10.1176/appi.ajp.162.5.890](https://www.zotero.org/google-docs/?vKWcPj)

[Carney, F. L. (1977). Outpatient Treatment of the Aggressive Offender. *American Journal of Psychotherapy*, *31*(2), 265–274. https://doi.org/10.1176/appi.psychotherapy.1977.31.2.265](https://www.zotero.org/google-docs/?vKWcPj)

[Colli, A., & Ferri, M. (2015). Patient personality and therapist countertransference. *Current Opinion in Psychiatry*, *28*(1), 46–56. https://doi.org/10.1097/YCO.0000000000000119](https://www.zotero.org/google-docs/?vKWcPj)

[Crandal, B. R., Foster, S. L., Chapman, J. E., Cunningham, P. B., Brennan, P. A., & Whitmore, E. A. (2015). Therapist perception of treatment outcome: Evaluating treatment outcomes among youth with antisocial behavior problems. *Psychological Assessment*, *27*(2), 710–725. https://doi.org/10.1037/a0038555](https://www.zotero.org/google-docs/?vKWcPj)

[De Page, L., Boulanger, M., De Villers, B., Di Virgilio, P., Pham, T., Saloppé, X., & Thiry, B. (2021). Countertransference in Forensic Inpatient Settings: An Empirical Examination of Therapist Responses to Patients With Psychotic Disorders. *Journal of Forensic Nursing*, *17*(1), 52–60. https://doi.org/10.1097/JFN.0000000000000308](https://www.zotero.org/google-docs/?vKWcPj)

[DeSorcy, D. R., Olver, M. E., & Wormith, J. S. (2020). Working Alliance and Psychopathy: Linkages to Treatment Outcome in a Sample of Treated Sexual Offenders. *Journal of Interpersonal Violence*, *35*(7–8), 1739–1760. https://doi.org/10.1177/0886260517698822](https://www.zotero.org/google-docs/?vKWcPj)

[Di Virgilio, P., De Page, L., & Titeca, P. (2021). Countertransference in Forensic Patients with Psychosis: Associations with Symptomatology, Inpatient Violence, and Psychopathic Personality Traits. *Journal of Forensic Psychology Research and Practice*, 1–16. https://doi.org/10.1080/24732850.2021.1874975](https://www.zotero.org/google-docs/?vKWcPj)

[Freestone, M. C., Wilson, K., Jones, R., Mikton, C., Milsom, S., Sonigra, K., Taylor, C., & Campbell, C. (2015). The Impact on Staff of Working with Personality Disordered Offenders: A Systematic Review. *PLOS ONE*, *10*(8), e0136378. https://doi.org/10.1371/journal.pone.0136378](https://www.zotero.org/google-docs/?vKWcPj)

[Gerbrandij, J. (2022). *The Impact of Psychopathy and Therapeutic Alliance on Treatment Outcome in a Dutch Forensic Treatment Sample* [Fordham University]. https://research.library.fordham.edu/dissertations/AAI29326347](https://www.zotero.org/google-docs/?vKWcPj)

[Graham, S. A. (1980). Psychotherapists’ attitudes toward offender clients. *Journal of Consulting and Clinical Psychology*, *48*(6), 796–797. https://doi.org/10.1037/0022-006X.48.6.796](https://www.zotero.org/google-docs/?vKWcPj)

[Kazdin, A. E., Marciano, P. L., & Whitley, M. K. (2005). The Therapeutic Alliance in Cognitive-Behavioral Treatment of Children Referred for Oppositional, Aggressive, and Antisocial Behavior. *Journal of Consulting and Clinical Psychology*, *73*(4), 726–730. https://doi.org/10.1037/0022-006X.73.4.726](https://www.zotero.org/google-docs/?vKWcPj)

[Kazdin, A. E., Whitley, M., & Marciano, P. L. (2006). Child–therapist and parent–therapist alliance and therapeutic change in the treatment of children referred for oppositional, aggressive, and antisocial behavior. *Journal of Child Psychology and Psychiatry*, *47*(5), 436–445. https://doi.org/10.1111/j.1469-7610.2005.01475.x](https://www.zotero.org/google-docs/?vKWcPj)

[Knaus, S., Grassl, R., Seidman, C., Seitz, T., Karwautz, A., & Löffler-Stastka, H. (2016). Psychiatrists’ emotional reactions: Useful for precise diagnosis in adolescence? *Bulletin of the Menninger Clinic*, *80*(4), 316–325. https://doi.org/10.1521/bumc.2016.80.4.316](https://www.zotero.org/google-docs/?vKWcPj)

[Lingiardi, V., Tanzilli, A., & Colli, A. (2015). Does the severity of psychopathological symptoms mediate the relationship between patient personality and therapist response? *Psychotherapy (Chicago, Ill.)*, *52*(2), 228–237. https://doi.org/10.1037/a0037919](https://www.zotero.org/google-docs/?vKWcPj)

[Michaud, L., Ligier, F., Bourquin, C., Corbeil, S., Saraga, M., Stiefel, F., Séguin, M., Turecki, G., & Richard-Devantoy, S. (2020). Differences and similarities in instant countertransference towards patients with suicidal ideation and personality disorders. *Journal of Affective Disorders*, *265*, 669–678. https://doi.org/10.1016/j.jad.2019.11.115](https://www.zotero.org/google-docs/?vKWcPj)

[Polaschek, D. L. L., & Ross, E. C. (2010). Do early therapeutic alliance, motivation, and stages of change predict therapy change for high‐risk, psychopathic violent prisoners? *Criminal Behaviour and Mental Health*, *20*(2), 100–111. https://doi.org/10.1002/cbm.759](https://www.zotero.org/google-docs/?vKWcPj)

[Rossberg, J. I., Karterud, S., Pedersen, G., & Friis, S. (2007). An empirical study of countertransference reactions toward patients with personality disorders. *Comprehensive Psychiatry*, *48*(3), 225–230. https://doi.org/10.1016/j.comppsych.2007.02.002](https://www.zotero.org/google-docs/?vKWcPj)

[Stefana, A., Bulgari, V., Youngstrom, E. A., Dakanalis, A., Bordin, C., & Hopwood, C. J. (2020). Patient personality and psychotherapist reactions in individual psychotherapy setting: A systematic review. *Clinical Psychology & Psychotherapy*, *27*(5), 697–713. https://doi.org/10.1002/cpp.2455](https://www.zotero.org/google-docs/?vKWcPj)

[Tanzilli, A., Gualco, I., Baiocco, R., & Lingiardi, V. (2020). Clinician Reactions When Working with Adolescent Patients: The Therapist Response Questionnaire for Adolescents. *Journal of Personality Assessment*, *102*(5), 616–627. https://doi.org/10.1080/00223891.2019.1674318](https://www.zotero.org/google-docs/?vKWcPj)

[Tanzilli, A., Trentini, C., Grecucci, A., Carone, N., Ciacchella, C., Lai, C., Sabogal-Rueda, M. D., & Lingiardi, V. (2022). Therapist reactions to patient personality: A pilot study of clinicians’ emotional and neural responses using three clinical vignettes from in treatment series. *Frontiers in Human Neuroscience*, *16*, 1037486. https://doi.org/10.3389/fnhum.2022.1037486](https://www.zotero.org/google-docs/?vKWcPj)

[Thompson, D. F., Ramos, C. L., & Willett, J. K. (2014). Psychopathy: Clinical features, developmental basis and therapeutic challenges. *Journal of Clinical Pharmacy and Therapeutics*, *39*(5), 485–495. https://doi.org/10.1111/jcpt.12182](https://www.zotero.org/google-docs/?vKWcPj)

[Treatment Outlines for Antisocial Personality Disorder: The Quality Assurance Project. (1991). *Australian & New Zealand Journal of Psychiatry*, *25*(4), 541–547. https://doi.org/10.3109/00048679109064448](https://www.zotero.org/google-docs/?vKWcPj)

[Walton, A., Jeglic, E. L., & Blasko, B. L. (2018). The Role of Psychopathic Traits in the Development of the Therapeutic Alliance Among Sexual Offenders. *Sexual Abuse*, *30*(3), 211–229. https://doi.org/10.1177/1079063216637859](https://www.zotero.org/google-docs/?vKWcPj)
